# INTERGENERATIONAL VERSUS PEER-LEARNING IN OLDER VOLUNTEER TRAINING: A MIXED-METHODS EVALUATION

**DOI:** 10.1093/geroni/igae098.4227

**Published:** 2024-12-31

**Authors:** Xinxin Cai, Nianzi Sun, Xue Bai

**Affiliations:** The Hong Kong Polytechnic University, Hung Hom, Kowloon, Hong Kong; The Hong Kong Polytechnic University, Hung Hom, Kowloon, Hong Kong; The Hong Kong Polytechnic University, Hung Hom, Kowloon, Hong Kong

## Abstract

Intergenerational learning activities enhance older adults’ well-being and intergenerational relationships, but their impact on learning outcomes is less understood. This explanatory sequential mixed-methods study evaluates a volunteer training program conducted between March and December 2023. Older adults were assigned to either a peer-learning group (n = 73) or an intergenerational learning group (n = 49), the latter including undergraduate students participating alongside them in a two-stage program: 1) thematic training workshops on family communication, later-life planning, or healthy living, and 2) designing and organizing a service project. Quantitative assessments were conducted before and after the workshops (T0, T1) and after the volunteer activities (T2). Outcomes included self-rated volunteering abilities, thematic knowledge acquisition, well-being (loneliness, happiness, life control), and intergenerational and peer relationships. After the program, 12 participants attended two focus group interviews. Repeated measures ANOVA revealed significant improvements in volunteer abilities and thematic knowledge only in the peer-learning group. A significant between-group effect was observed for thematic knowledge. MANOVA results indicated a significant time*group interaction, with the intergenerational group showing reduced loneliness. Qualitative analysis suggested older adults were highly motivated to learn and contribute to the community. While they valued interaction with younger generations, they did not perceive it as influencing their learning. However, they believed student commitment influenced their volunteer experiences. This study highlights the learning capabilities of older adults in delivering skill-based volunteer activities. While intergenerational components may not directly enhance learning outcomes, they can improve well-being. Their inclusion should align with program goals for maximum impact.

